# Preferences of the Public for Sharing Health Data: Discrete Choice Experiment

**DOI:** 10.2196/29614

**Published:** 2021-07-05

**Authors:** Jennifer Viberg Johansson, Heidi Beate Bentzen, Nisha Shah, Eik Haraldsdóttir, Guðbjörg Andrea Jónsdóttir, Jane Kaye, Deborah Mascalzoni, Jorien Veldwijk

**Affiliations:** 1 Centre for Research Ethics & Bioethics Department of Public Health and Caring Sciences Uppsala Universitet Uppsala Sweden; 2 Norwegian Research Center for Computers and Law Faculty of Law University of Oslo Oslo Norway; 3 Centre for Health, Law, and Emerging Technologies Faculty of Law University of Oxford Oxford United Kingdom; 4 Social Science Research Institute University of Iceland Reykjavik Iceland; 5 Centre for Health, Law and Emerging Technologies Melbourne Law School University of Melbourne Melbourne Australia; 6 Institute for Biomedicine Bolzano Italy; 7 Erasmus School of Health Policy and Management Erasmus University Rotterdam Netherlands

**Keywords:** preferences, discrete choice experiment, health data, secondary use, willingness to share

## Abstract

**Background:**

Digital technological development in the last 20 years has led to significant growth in digital collection, use, and sharing of health data. To maintain public trust in the digital society and to enable acceptable policy-making in the future, it is important to investigate people’s preferences for sharing digital health data.

**Objective:**

The aim of this study is to elicit the preferences of the public in different Northern European countries (the United Kingdom, Norway, Iceland, and Sweden) for sharing health information in different contexts.

**Methods:**

Respondents in this discrete choice experiment completed several *choice tasks*, in which they were asked if data sharing in the described hypothetical situation was acceptable to them. Latent class logistic regression models were used to determine attribute-level estimates and heterogeneity in preferences. We calculated the relative importance of the attributes and the predicted acceptability for different contexts in which the data were shared from the estimates.

**Results:**

In the final analysis, we used 37.83% (1967/5199) questionnaires. All attributes influenced the respondents’ willingness to share health information (*P*<.001). The most important attribute was whether the respondents were informed about their data being shared. The possibility of opting out from sharing data was preferred over the opportunity to consent (opt-in). Four classes were identified in the latent class model, and the average probabilities of belonging were 27% for class 1, 32% for class 2, 23% for class 3, and 18% for class 4. The uptake probability varied between 14% and 85%, depending on the least to most preferred combination of levels.

**Conclusions:**

Respondents from different countries have different preferences for sharing their health data regarding the value of a review process and the reason for their new use. Offering respondents information about the use of their data and the possibility to opt out is the most preferred governance mechanism.

## Introduction

### Background

Digital technological development in the last 20 years has led to significant growth in digitally collecting, using, and sharing health data. This is partly due to the development and adoption of electronic medical records, genotyping, biobanking, and self-tracking applications via mobile devices. Different domains, such as health care, medical research, and technological and pharmaceutical companies, have become increasingly dependent on collecting and sharing data digitally to develop health care and new medical and technological products [1-3]. It has also led individuals to take a more active role in seeking out health information, thus managing and promoting their own health by having access to new health websites and mobile apps [4,5].

As different domains are dependent on public data, it is important to maintain public trust in the digital world. There is growing literature about preferences of the public, research participants, and patients for data sharing. People’s willingness to share data for secondary use is dependent on contextual factors such as the type of data being linked, level of identification, and the new purpose for the data being shared [6-9]. A study that investigated the public’s preferences regarding data linkage for health research showed that the type of information shared is the most important factor for people deciding whether they are willing to consent to the new use of their data [10]. Other studies show that people are interested in sharing their health information to improve health but are less willing to make data available to companies and insurance companies whose purpose for using the data may be unclear or not align with the public’s expectations [7,11,12].

Previous research on people’s willingness to share health data digitally has focused on one particular factor, such as the purpose of data sharing [6,7]. In addition, these studies in this area have been constrained by a specific context, such as looking at data movement within a health care setting [8,13,14]. To our knowledge, no studies have investigated how individuals’ preferences change depending on the context in which health data are used, what type of information is involved, which different control mechanisms are considered appropriate for different contexts, and how an individual’s acceptance of sharing data might change in response to changing contexts. There is a lack of knowledge on individuals’ trade-off behavior in the current situation where data are linked across fields. Such studies are needed to provide a comprehensive understanding of the trade-offs between different factors, thereby informing policymaking and legal development therein [15].

We would like to evoke the need for a stated choice method that investigates behavioral intention to share health information, which is captured through trade-offs between varying levels, such as who the new user of the data is and for what reason the data will be shared. It is necessary to move away from the problematic single (fixed) scenario that captures people’s behavioral intentions using Likert scales [15,16]. A stated choice method such as discrete choice experiment (DCE) require respondents to make a decision when the circumstances change. This method provides a deep understanding of context-specific factors that people value. Moreover, using DCE as a method aligns with the theory by philosopher Helen Nissenbaum, which emphasizes that privacy is perceived and expected differently, depending on the norms and values surrounding the context [17].

### Objective

The aim of this study is to elicit the public’s preferences and the heterogeneity in preferences in different Northern European countries (Sweden, Norway, Iceland, and the United Kingdom) to share health information in different contexts in order to determine what governance structures should be in place in the health sphere.

## Methods

### Discrete Choice Experiment

The DCE method is increasingly used in health care fields to quantify the preferences of specific target populations concerning any health-related product or service [18-20]. In a DCE, respondents are asked to complete several *choice tasks*. Each choice task describes the situation at hand. The description of the situation is based on its characteristics or *attributes* with systematically varying levels. In our case, respondents were asked to choose, to accept, or reject a situation several times. By monitoring their decisions in each choice task, their preferences were elicited. DCEs draw upon *random utility theory*, according to which an individual derives a certain *utility* for what the individual is confronted with in a choice task [21-24]. By comparing the attribute-level estimates, conclusions can be drawn about the importance of the attributes relative to each other. Moreover, the utility and acceptability of different data sharing situations can be calculated based on the attribute-level estimates from the experiment.

### DCE Development

The salient factors of digital health data sharing were identified through a three-step approach [25]. First, a literature review was performed to identify the possible factors that influence respondents’ willingness to share their health data. Second, based on the output of the literature review, 14 focus group discussions were conducted with members of the public in the United Kingdom, Iceland, and Sweden. We carried out a comparative investigation of the respondents’ attitudes, expectations, and beliefs about sharing health data. Focus group participants from all three countries mentioned the following factors as important when allowing data to be shared: level of identification, the reason for the new use, type of information being shared, the data subject being informed, and the monitoring of sharing. After the focus group discussion, a nominal group technique was used to ask participants to rank the importance of the different aspects or factors discussed in the focus groups, in addition to an a priori list of factors identified from the prior literature review. During the nominal group technique session, participants were asked to rank the potential factors from most to least important and then discuss them in the group. The authors and content experts thoroughly discussed the attributes and levels to confirm their relevance. On the basis of these steps, seven attributes were selected (Textbox 1). During a two-hour webinar, content experts were asked to comment on the attributes as well as framing of the levels. Eight think-aloud interviews [26] were conducted (four in Sweden, two in Iceland, and two in the United Kingdom) to evaluate whether correct wording was used and whether the target population understood the attributes, levels, educational information, and choice tasks. Finally, a two-day workshop was held where both method and content experts were invited to reach a consensus on chosen attributes and levels. Areas of expertise included law, philosophy, ethics, social science, and stated preference research.

**List of all attributes and levels included in the final discrete choice experiment.**

**Attributes and Levels**

1. Health information collector: different collectors can collect health information. The different collectors are as follows:
  - A technological company with which you have used a service, program, or application for your phone or computer. You may have used a service through the company’s website, where you have entered information about yourself. Alternatively, you have downloaded an app to your phone, and it has collected information about your health.
  - An academic research project where you have participated and they have collected health information about you.
  - Your health care provider (hospital or general practitioner) who has collected health information about you regarding your care.
2. Data user: your health information will be shared to a new data user. This new recipient may be: 
  - A technological company that develops health app which can be used to predict diagnoses.
  - A pharmaceutical company that develops and manufactures new medicines.
  - An academic research project that produces new knowledge by testing hypotheses and theories about human health.
  - A national authority, for example, the public health authority or information and commissioner’s office, which is responsible for the health of the population. They can track peoples’ health through population registers to prevent disease.
3. The reason of data use: this aspect describes the reason why the data user wants to have access to your health information. The different reasons may be:
  - Develop a new product or service. It can be a medical device, a drug, or app for your phone, or a new health service or program.
  - Promote, advertise, or market their product or service to personalize communication. For direct advertising to a specific target group for a new service or product.
  - Investigate a policy initiative. Your health information can provide a basis for a new policy initiative at a national level. It may be to improve services for a specific part of the population or to identify new preventive measures to improve public health.
  - Evaluate the quality of the data user’s product or service, and planning resource distribution in the future.
4. Information and consent: this aspect is about whether you will be informed if your health information is being shared.
  - You will not be informed that health information about you is being shared and used in a new context.
  - You will be informed that health information about you is being shared and used in a new context.
  - You will be informed that health information about you is being shared and used in a new context as well as be told that you can opt out.
  - You will be informed and asked to consent that health information about you is being shared and used in a new context.
5. Review of data sharing: before your data are shared, there might be a review of the reason and how the data user will store and use your health information. The data user needs to apply for access to the health information. The reviewer makes a decision based on national law.
  - There will be no review of the data sharing.
  - A committee will review the transfer of your health information to the new context. 
  - A committee will review the transfer and the use of your health information in the new context.

A Bayesian D-efficient design was used for this DCE to strive for reliable parameter estimates [21,27,28]. The design was developed using NGene (version1.2.1; ChoiceMterics 2012). This is the most commonly used design strategy and is congruent with the guidelines of the International Society of Pharmacoeconomics and Outcome Research on good research practice [27]. Pilot testing priors based on best guesses were used to inform the design using 500 Halton draws and 1000 repetitions. For this design, we assumed that there would be no interaction between attributes. The level balance (ie, all levels appearing an equal number of times) was optimized. The pilot design had a D-error of 0.31. A total of 28 unique choice tasks were generated and divided into four blocks. Respondents were randomly assigned to either block and answered seven choice tasks.

We pilot tested the draft questionnaire among our target population (n=50) in each of the four countries. The attribute-level estimates that significantly contributed to the choice from the pilot study served as direct prior input for the design of the final DCE questionnaire.

### Questionnaire

The questionnaire consisted of three parts. The first part contained questions regarding demographic characteristics (eg, age, gender, educational level, self-reported health status, and long-term health conditions). The eHealth Literacy Scale is designed to assess people’s perceived skills at using information technology for health [29], and it comprises eight items assessing different aspects of eHealth literacy (eHL). Each item had five response categories: strongly disagree, disagree, neither agree nor disagree, agree, and strongly agree.

**Figure 1:**
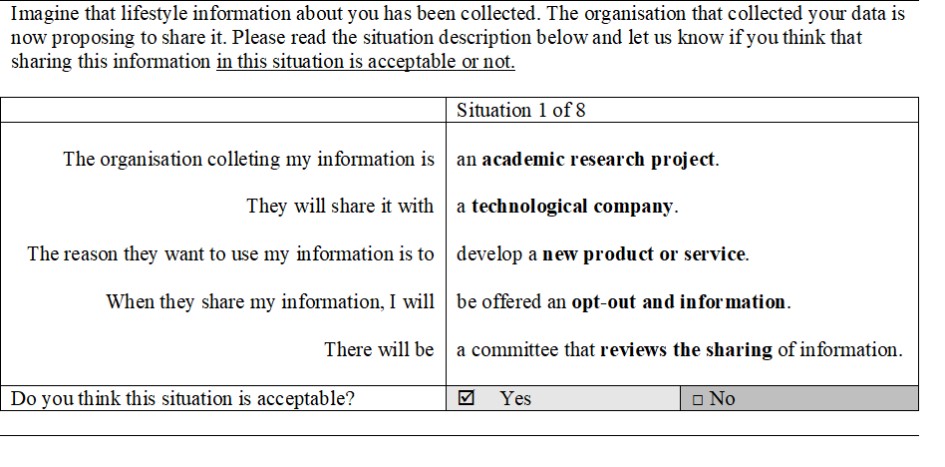
An example of a discrete choice experiment with one choice situation.

The second part was the DCE. Each participant was given an alternative choice that they were asked to accept or reject. An additional level was added to the attribute *Information and consent* in the final design. Therefore, the final DCE consisted of 32 unique choice tasks divided into four blocks, and each participant answered eight choice tasks for two types of health information (16 choice tasks). Before respondents were asked to complete the choice tasks, they received detailed information on the meaning of all attributes and levels, as well as an example of how to complete a choice task. This particular DCE topic describes a situation. It is not tradable in the ordinary sense (one product or service over another). Given that this topic is not tradable like regular DCE, each participant was given a choice alternative where the participant was asked to accept or reject. Figure 1 shows a choice task with one situation. The remaining attributes changed in a systematic manner between the different levels.

The third part of the questionnaire related to trust in different domains and other people, attitudes toward new technology, and self-assessed eHL.

### Study Population

Ethics approval was obtained before the start of the study from the Ethical Review Boards in the countries where this was required (University of Oxford Central University Ethics Committee REF: R63378/RE002; Swedish Ethical Review Authority Dnr 2020-00623; University of Iceland Science Ethics Committee VSH-2019-019).

The DCE was web-based, and respondents were invited to participate via the recruitment service SurveyEngine [30]. The recruitment company performed opt-in survey panels in Sweden, Norway, and the UK. The respondents decided on incentive models that worked best for their specific membership, such as cash, vouchers, virtual currencies, points for gift cards, or participation in raffles. The respondents received €1.50 (US $1.80) for answering our survey. The Icelandic respondents were randomly selected from the Social Science Research Institute’s Online Panel at the University of Iceland. Respondents in the Social Science Research Institute Online Panel were recruited through random samples drawn from the National Population Register in Iceland. A lottery to win one of the two gift vouchers of €65 (US $77.40) was used as an incentive.

We aim to obtain a representative sample of the general population of each country in terms of gender and age. Data were collected from August to November 2020.

### Statistical Analysis

#### Descriptive Statistics

Descriptive statistics (means and frequencies) were used to summarize all the variables of interest. The overall level of eHL was calculated for each respondent. Individuals responding strongly disagree or disagree to one of the items were categorized as having inadequate eHL. Individuals responding with neither agree nor disagree with one of the items were categorized as having problematic eHL. Individuals responding agree or strongly agree to all the items were categorized as having sufficient eHL.

One-way analysis of variance and nonparametric measures were used to test the differences between the personal characteristics of each country.

#### Preferences for Sharing Health Information

The discrete choice data collected in the survey were first analyzed separately for each country using a binary logit model. The Swait-Louviere test was performed to investigate whether there were significant scale differences across samples from different countries [31]. Latent class models were then used to determine the attribute-level estimates and importance weights of the attributes. The latent class model identifies classes of respondents based on unobserved (latent) heterogeneity in preferences [32]. Akaike information criteria and log-likelihood were used to determine the best-fitting model [33]. All attributes were effect-coded [34], meaning that the reference category was coded as −1, and the sum of all the coded levels for each attribute was zero. A constant term was also estimated to quantify the utility associated with rejecting information sharing under the presented situation (Intercept). All results were considered statistically significant at *P*<.05.

The final utility function was as follows:

U = V_rta&b|c_ + ε = β0|c * reject_rta&b|c_ + β1 * collector_technological_rta&b|c_ + β2 * collector_research_rta&b|c_ + β3 * user_technological_rta&b|c_ + β4 * user_pharmaceutical_rta&b|c_ + β5 * user_research_rta&b|c_ + β6 * reason_develop_rta&b|c_ + β7 * reason_promoting_rta&b|c_ + β8 * reason_policy_rta&b|c_ + β9 * information_not informed_rta&b|c_ + β10 * information_informed_rta&b|c_ + β11 * information_opt-out_rta&b|c_ + β12 * review_no review_rta&b|c_ + β13 * review_sharing_rta&b|c_ + ε

**(1)**

V is the observed utility of accepting to share health data with a second user based on what respondents *r* belonging to class *c* reported for the alternative *a* in choice task *t*. The *β*_0_ represents the alternative specific constant, and *β*_1_-*β*_13_ are attribute-level estimates that indicate the relative importance of each attribute level. Data cleaning and descriptive statistics were performed using R (version 4.0.2; R Core Team). The latent class logistic regression was performed with the econometric software NLogit 5.0 (Econometric Software, Inc), using 100 random draws. In latent class analysis, unobserved preference heterogeneity among respondents is modeled as discrete classes with similar preferences or choice patterns but with different variances across classes [35,36]. As the probability of a participant belonging to any specific class cannot be directly observed, the model searches for groups of respondents sharing similar choice patterns. Once choice patterns have been stratified into classes, the model could determine the probability of a participant with certain characteristics being assigned to each class (class assignment model). This separate logit model was fitted to determine the associations between individual class membership and country. We also explored potential associations with other variables, such as age, sex, and E-HL. When individually added to the model, they all significantly contributed to latent class assignment. However, when adding multiple covariates into a one-class assignment model, we observed multicollinearity between the variables. As *country* was the most important variable for this overall study (and a necessity to include as we pooled data from multiple countries into one data set), we focused on that variable in this study. We will explore the impact of other variables separately in the analysis conducted on data from separate countries to avoid this collinearity caused by country differences.

#### Relative Importance of the Attributes

Using the relative preference weights, that is, the attribute-level estimates from the DCE, we calculated the relative importance of the attributes. For each attribute, the total impact on utility was determined by subtracting the lowest from the highest estimate within each attribute. All attributes were divided according to the highest difference value. This provided a relative distance between the most important attributes and all other attributes.

#### Acceptance Uptake

The acceptance uptake (also referred to as predicted probability [37], participation probability [38], predicted uptake [24,39], or subsequent uptake [40]) was calculated for different scenarios for sharing health data. This was determined for different potential scenarios and could inform future implementation strategies. Acceptance uptake can be understood as the probability that a participant would choose the described scenarios; alternatively, the number of respondents out of 100 that would accept the scenarios described. These scenarios represent existing or hypothetical scenarios. Using the attribute levels, scenarios based on specific data sharing and governance features were assembled. The utility for a specific scenario is calculated by using the following equation:

V_Scenario 1_ = β_A_ + β_B_ + β_C_

**(2)**

The acceptance uptake, the probability of accepting, was then calculated by using the following equation:

Acceptance uptake = 1/(1+exp^-V_Scenario 1_^)

**(3)**

## Results

### Respondents’ Characteristics

In total, 5199 respondents answered the questionnaire (Sweden, n=1208; Norway, n=928; Iceland, n=2187; United Kingdom, n=876). Respondents who completed the survey in less than 5 minutes (n=97) or did not complete the entire survey (n=3135) were excluded. In the final analysis, we used 37.83% (1967/5199) of the questionnaires. The mean ages of the respondents were 50.4 years (SD 16.9) in Sweden, 48.3 years (SD 17.2) in Norway, 49.9 years (SD 15.9) in the United Kingdom, and 48.2 years (SD 17.2) in Iceland. Respondents with university education included 36.2% (162/447) in Sweden, 39.3% (167/425) in Norway, 52.1% (232/445) in the United Kingdom, and 57.3% (287/501) in Iceland. Respondents with sufficient eHL included 30.6% (137/447) in Sweden, 22.8% (97/425) in Norway, 36.6% (163/445) in the United Kingdom, and 20.6% (103/501) in Iceland. The respondents’ characteristics are presented in Tables 1-3.

**Table 1:** Descriptive statistics of the respondents presented as percentages, mean, or median with statistical testing between the different countries.

Variates			Sweden (n=481)		Norway (n=465)		United Kingdom (n=477)		Iceland (n=544)		*P* value (ANOVA^a^)	
**Age (years)**												.15
	Mean (SD)	50.3 (16.9)		48.1 (17.2)		49.6 (15.9)		48.3 (17.2)				
	Median (range)	53 (18-88)		50 (18-84)		49 (18-90)		47 (19-88)				
Survey duration, mean (SD)			15.5 (11.5)		15.4 (10.2)		12.8 (7.36)		20.7 (14.9)		<.001	

^a^ANOVA: analysis of variance.

**Table 2:** Descriptive statistics of the respondents presented as percentages with Chi-square testing between the different countries.

Variates		Sweden (n=438)	Norway (n=424)	United Kingdom (n=450)	Iceland (n=538)	*P* value (Chi-square test)	
**Gender, n (%)**							.79
	Female	219 (50)	226 (53.3)	236 (52.4)	268 (49.8)		
	Male	218 (49.8)	197 (46.5)	214 (47.6)	268 (49.8)		
	Other	1 (0.2)	1 (0.2)	0 (0)	2 (0.4)		
**General health status, n (%)**							<.001
	Good	296 (67.6)	285 (67.2)	335 (74.4)	445 (82.7)		
**Chronic health condition, n (%)**							<.001
	No	203 (46.3)	179 (42.2)	262 (58.2)	304 (56.5)		

**Table 3:** Descriptive statistics of the respondents presented as percentages with Kruskal-Wallis testing between the different countries.

Variates		Sweden (n=447)	Norway (n=425)	United Kingdom (n=445)	Iceland (n=501)	*P* value (Kruskal-Wallis test)	
**Highest educational level, n (%)**							<.001
	High school	251 (56.2)	234 (55.1)	129 (29)	181 (36.1)		
	Primary school	34 (7.6)	24 (5.6)	84 (18.9)	33 (6.6)		
	University	162 (36.2)	167 (39.3)	232 (52.1)	287 (57.3)		
**eHealth literacy, n (%)**							<.001
	Insufficient	115 (25.7)	126 (29.6)	116 (26.1)	183 (36.5)		
	Problematic	195 (43.6)	202 (47.5)	166 (37.3)	215 (42.9)		
	Sufficient	137 (30.6)	97 (22.8)	163 (36.6)	103 (20.6)		
**How often they are using apps related to health, n (%)**							<.001
	Daily	64 (14.3)	71 (16.7)	107 (24)	87 (17.4)		
	Weekly	52 (11.6)	44 (10.4)	51 (11.5)	69 (13.8)		
	Monthly or more seldom	121 (27.1)	152 (35.8)	57 (12.8)	145 (28.9)		
	Never	176 (39.4)	109 (25.6)	212 (47.6)	144 (28.7)		
	I don’t know	34 (7.6)	49 (11.5)	18 (4)	56 (11.2)		
**Internet is useful, n (%)**							.04
	Yes	312 (69.8)	261 (61.4)	306 (68.8)	341 (68.1)		
**Internet is an important source for health information, n (%)**							.05
	Yes	365 (81.7)	328 (77.2)	330 (74.2)	395 (78.8)		

### Preferences for Sharing Health Information

The coefficients for all attributes were statistically significant and had signs consistent with our expectations in the binary logit model (Table 4). The respondents found situations such as when the collector was their health care provider, if the new user was a national authority, and the reason was to evaluate the quality of the care as being more acceptable. Moreover, respondents thought it was important to be informed and preferred situations that offered the opportunity to opt out, and that there was a review of the sharing and use of the health information in place.

**Table 4:** Estimates for the multinomial logit model with all countries together.

Attribute and level		Logit		
		Estimate (SE)	*P* value	95% CI
**Collector**				
	A technological company	−0.19 (0.02)	<.001	−0.22 to −0.16
	A research project	0.08 (0.02)	<.001	0.05 to 0.11
	Your health care provider (Ref^a^)	0.11 (N/A^b^)	N/A	N/A
**New user**				
	A technological company	−0.26 (0.02)	<.001	−0.31 to −0.22
	A pharmaceutical company	−0.03 (0.02)	.15	−0.08 to 0.012
	A research project	0.12 (0.02)	<.001	0.08 to 0.16
	A national authority (Ref)	0.17 (N/A)	N/A	N/A
**Reason**				
	Develop a new product or service	0.15 (0.02)	<.001	0.11 to 0.20
	Promoting, advertising, or marketing	−0.47 (0.02)	<.001	−0.52 to −0.42
	Investigate a policy initiative	0.11 (0.02)	N/A	0.07 to 0.15
	Evaluate the quality (Ref)	0.21 (N/A)	N/A	N/A
**Information**				
	Not informed	−0.90 (0.02)	<.001	−0.95 to −0.85
	Informed	−0.08 (0.02)	.001	−0.12 to −0.03
	Informed and ability to opt out	0.51 (0.02)	<.001	0.46 to 0.55
	Informed and consent (Ref)	0.47 (N/A)	N/A	N/A
**Reviewing**				
	No specific review	−0.52 (0.02)	<.001	−0.56 to −0.49
	Review of sharing	0.25 (0.02)	<.001	0.22 to 0.30
	Review of sharing and use (Ref)	0.27 (N/A)	N/A	N/A
Intercept		0.51 (0.01)	<.001	0.49 to 0.54

^a^Reference category.

^b^N/A: not applicable.

Four classes were identified as providing the best fit in the latent class model (Table 5). The information criteria suggested a significant improvement in the fit for the latent class specification over the binary model. All attributes were statistically significant in all classes (besides the new user for class 3), which means that all attributes influenced the decision to accept or reject health information being shared.

**Figure 2:**
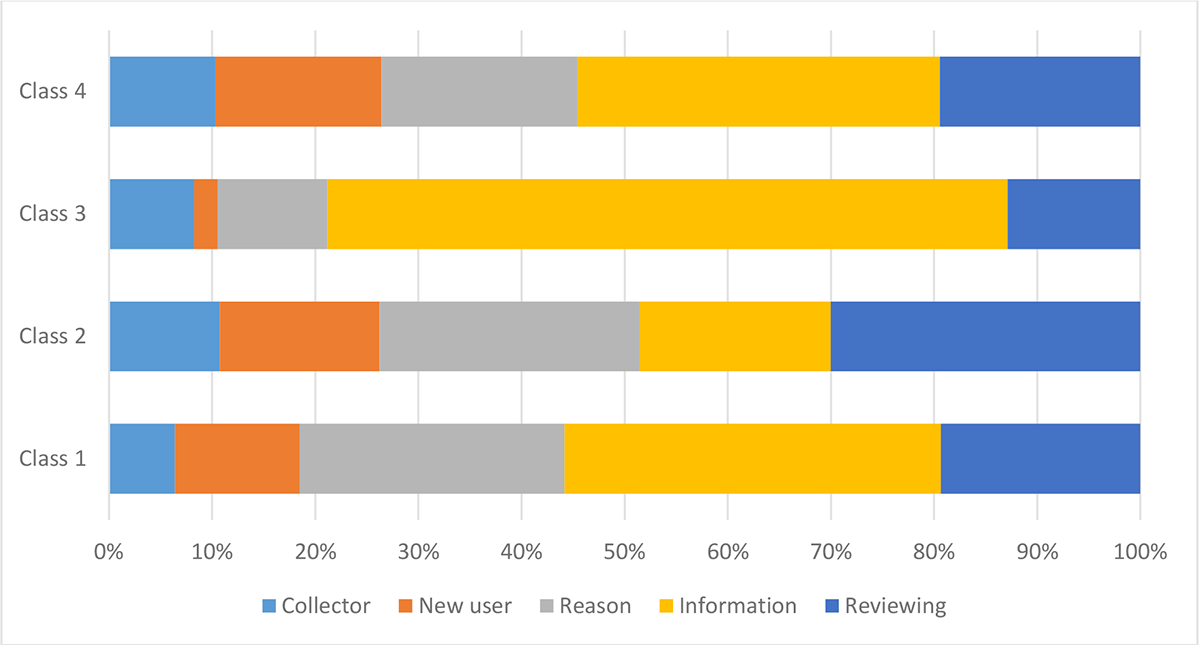
Relative importance score for respondents’ preferences stratified using the four-class model. The reason and having a review process in place were most important for class 2. Being informed was most important for classes 1, 3, and 4.

The average probability of belonging to class 1 was 27%, class 2 was 32%, class 3 was 23%, and class 4 was 18%. The four classes displayed some important differences. The intercept term, which reflects the average utility associated with the rejection option, was positive and significant in the binary model (0.51; Table 4). This finding suggests that, on average, respondents in this study preferred *not* *to* *share* their health data. The intercept term in the latent class model was negative in class 4, which suggests that class 4 was positive for sharing data. Classes 1, 3, and 4 found a review process regarding the use to be insufficient, whereas class 2 did not (Figure 2).

**Table 5:** Estimates for the latent class model, four classes with country as class membership.

Attribute and level			Latent class						
			Class 1		Class 2		Class 3		Class 4
			Estimate (SE)		Estimate (SE)		Estimate (SE)		Estimate (SE)
**Collector**									
	A technological company	−0.26^a^ (0.10)		−0.37^b^ (0.04)		−0.36^b^ (0.06)		−0.40^b^ (0.08)	
	A research project	0.14 (0.11)		0.15^b^ (0.03)		0.21^b^ (0.05)		0.16^a^ (0.07)	
	Your health care provider (Ref^c^)	0.13 (N/A^d^)		0.22 (N/A)		0.15 (N/A)		0.25 (N/A)	
**New user**									
	A technological company	−0.41^a^ (0.16)		−0.53^b^ (0.04)		−0.07 (0.07)		−0.59^b^ (0.09)	
	A pharmaceutical company	−0.12 (0.14)		−0.08 (0.04)		−0.08 (0.07)		−0.05 (0.08)	
	A research project	0.17 (0.13)		0.29^b^ (0.05)		0.07 (0.07)		0.23 (0.09)	
	A national authority (Ref)	0.36 (N/A)		0.32 (N/A)		0.08 (N/A)		0.41 (N/A)	
**Reason**									
	Develop a new product or service	0.56^b^ (0.16)		0.33^b^ (0.04)		0.04 (0.07)		0.43^b^ (0.09)	
	Promoting, advertising, or marketing	−1.06^b^ (0.19)		−0.98^b^ (0.06)		−0.41^b^ (0.09)		−0.76^b^ (0.10)	
	Investigate a policy initiative	0.10 (0.14)		0.25^b^ (0.04)		0.05 (0.07)		−0.05^b^ (0.09)	
	Evaluate the quality (Ref)	0.40 (N/A)		0.40 (N/A)		0.32 (N/A)		0.38 (N/A)	
**Information**									
	Not informed	−1.47^b^ (0.19)		−0.66^b^ (0.06)		−2.95^b^ (0.12)		−1.36^b^ (0.12)	
	Informed	0.02 (0.18)		−0.04 (0.05)		−0.41^b^ (0.07)		−0.11 (0.09)	
	Informed and ability to opt out	0.82^b^ (0.19)		0.35^b^ (0.05)		1.61^b^ (0.10)		0.84^b^ (0.08)	
	Informed and consent (Ref)	0.65 (N/A)		0.31 (N/A)		1.34 (N/A)		0.51 (N/A)	
**Reviewing**									
	No specific review	−0.78^b^ (0.13)		−1.07^b^ (0.05)		−0.58^b^ (0.07)		−0.75^b^ (0.09)	
	Review of sharing	0.43^b^ (0.12)		0.50^b^ (0.04)		0.28^b^ (0.06)		0.46^b^ (0.07)	
	Review of sharing and use (Ref)	0.35 (N/A)		0.57 (N/A)		0.30 (N/A)		0.29 (N/A)	
Intercept			3.60^b^ (0.14)		0.31^b^ (0.04)		0.84^b^ (0.06)		−2.01^b^ (0.09)
AIC^e^			29,730 (N/A)		N/A		N/A		N/A
Log-likelihood			−14,797 (N/A)		N/A		N/A		N/A
Average class probability (%)			27 (N/A)		32 (N/A)		23 (N/A)		18 (N/A)
**Class membership**									
	Constant	0.96^b^ (0.17)		0.73^b^ (0.19)		0.87^b^ (0.18)		Ref	
	Sweden	−0.29 (0.22)		0.09 (0.23)		−0.91^b^ (0.25)		Ref	
	Norway	−1.06^b^ (0.22)		−0.78^b^ (0.24)		−0.50^a^ (0.22)		Ref	
	Iceland	−0.76^b^ (0.22)		0.15 (0.23)		−0.98^b^ (0.24)		Ref	

^a^Significance at 5% level.

^b^Significance at 1% level.

^c^Ref: Reference category.

^d^N/A: not applicable.

^e^AIC: Akaike information criteria.

The country significantly predicted class membership as follows: Norwegians were more likely to think that being informed and knowing the purpose would be important when deciding on data sharing (class 1). Icelanders were more likely to think that a review of the sharing and knowing the purpose would be important when deciding on data sharing (class 2). Swedes were more likely to belong to classes 1 and 2. Respondents from the United Kingdom were divided evenly into all classes.

### Relative Importance of the Attributes

**Figure 3:**
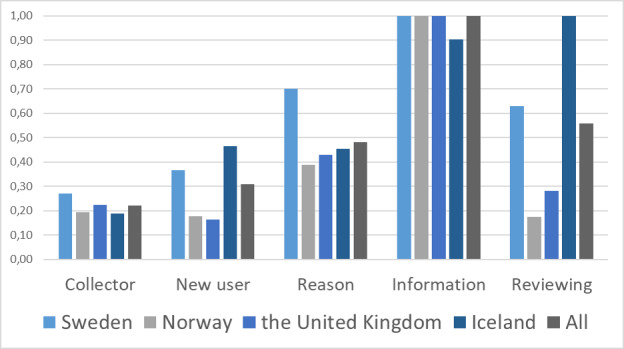
Relative importance score for all respondents’ preferences, stratified by country. Receiving information and having the opportunity to opt out was the most important attribute, on average, followed by review process and the reason for sharing the information.

In a situation where health data were about to be transferred to new users, respondents reported the importance of being informed. Having a review process in place was the second most important attribute (Figure 3). Swedish respondents placed more importance on the reason that their health information would be shared compared with respondents from Norway, the United Kingdom, and Iceland. Having a review process in place was the most important attribute for respondents in Iceland.

The type of shared information does not change the relative order of the attributes. However, the reason for sharing information was more important when genetic information was shared, as opposed to lifestyle information (Multimedia Appendix 1).

### Acceptance Uptake

The combination of levels that was most preferred gave an acceptance uptake of 85%, that is, health information collected by a health care provider, evaluating the quality of the national authority’s service, planning how resources should be distributed in the future, being informed, having the ability to opt out, with a review process of sharing and use of information. The least preferred combination gave 14% acceptance uptake, that is, health information collected by a technological company; promoting, advertising, marketing for a new technological company; not being informed of the sharing; and with no review process in place.

Depending on the attribute levels combined into new hypothetical scenarios in which data will be shared from a health care setting to a technological company, the uptake probability varies between 18% and 77% (Table 6). In situations where people were not informed about their data being transferred, the uptake probability increased if a review process was in place. For further scenarios, view Multimedia Appendix 2.

**Table 6:** The acceptance uptake (class adjusted probability) when health information is shared from a health care setting to a technological company.

Scenarios		Not informed (%)	Informed (%)	Opt-out (%)	Consent (%)
**No review**					
	Develop a new product or service	32	49	63	63
	Promoting, advertising, or marketing	18	37	53	60
	Investigate a policy initiative	29	47	61	61
	Evaluate the quality	32	51	64	64
**Review of use**					
	Develop a new product or service	45	65	77	76
	Promoting, advertising, or marketing	32	52	65	65
	Investigate a policy initiative	43	63	75	74
	Evaluate the quality	46	66	77	76
**Review of use and transfer**					
	Develop a new product or service	45	65	77	76
	Promoting, advertising, or marketing	31	53	65	64
	Investigate a policy initiative	43	63	74	73
	Evaluate the quality	46	66	77	76

## Discussion

### Principal Findings

This DCE study elicited preferences of citizens in Sweden, Norway, Iceland, and the United Kingdom for sharing health data digitally. Respondents in this study indicated that they preferred to share their data when a national authority was going to be the new user of the data. The second preferred new user was an academic research project. On average, and in almost all classes, the respondents preferred a pharmaceutical company as a new user, instead of a technological company. This might be because pharmaceutical companies are well regulated.

The findings show that, on average, respondents from these countries find it more acceptable if they are at least informed about the fact that data will be shared. In addition, having a review process in place to oversee the sharing and use of data was important to people, including the reason the new user had to request data to be shared. These findings provide evidence that supports the European Union General Data Protection Regulation (GDPR) 2016/679 [41], where transparency is one of the foundational principles and serves as a cornerstone of the Regulation. The GDPR advocates informational self-determination by increasing transparency requirements for data collection practices. It also strengthens individuals’ rights regarding their personal data. However, consent is only one of several lawful bases to process personal data listed in Articles 6 and 9 of the GDPR, and cannot be used for the sake of appearances; it is only a lawful base if the data subject is offered control and a genuine choice [42].

Even though all participating countries are required to adhere to the same regulation, the results of this study show that respondents in different countries value different factors when health information is shared. Our results indicate that having a review process in place can be more important for respondents in Sweden and Iceland. However, it can be practically and economically challenging to implement a review process, especially among all private companies. Moreover, having different governance mechanisms in each country can be problematic for cross-border sharing. Therefore, we emphasize the purpose limitation principle, Article 5(1)(b) GDPR [41], that the collection purposes shall be specified, explicit, and legitimate, and that the personal data shall not be further processed in a manner incompatible with those purposes. Respect for purpose limitation can meet peoples’ concerns and requests for contextual control and respect for expectations. This can make the difference between success and failure for the population’s acceptance of sharing [43].

It was hypothesized that respondents would prefer to share data if they were offered the opportunity to consent. However, the results show that respondents preferred an opportunity to opt out of the opportunity to consent. It might be enough to have the ability to opt out where other governance mechanisms are in place. From a learning health system perspective in countries with government-financed health care, such as the ones studied here, it might be easier to argue in favor of extensive data sharing between health care providers and medical researchers to account for the shared interest in improved health care for patients [44]. Other rights such as access to health care and quality of health care are also vital concerns [45].

Health care service providers need collaboration with technological companies to improve health via new technological products [46]. Therefore, it could be valuable to evaluate a *thick* opt-out procedure that will provide more participation, and simultaneously acknowledge people’s rights to decide over their own private sphere [47]. A *thick* opt-out, or an informed opt-out, in this context means that people become well informed that their data will be shared, but they will not actively agree to the sharing. The default position is that data will be shared and people who do not want to share their health information can actively disagree by opting out. Hence, we identified a need to investigate whether a *thick* opt-out procedure can be sufficient in some contexts. When calculating the acceptance uptake (Table 6), we found an even proportion of people accepting and rejecting their data being shared when offered an opportunity to consent rather than opting out. To account for the range of governance preferences of individuals, dynamic, and meta-consent models [48,49], which allow individuals to first choose their preferred governance model, ought to be studied in the context of not only medical research but also, more generally, for sharing health data in society.

Collecting, storing, and sharing health data is now part of our society. A study by Xafis [50] found that many respondents were of the opinion that data that cannot be traced back to an individual holds a different status compared with identifiable data and could be used without consent. Most respondents in our study preferred to be informed and had the opportunity to opt out or consent to the transfer, even when the data in question would be processed anonymously. This is not required by GDPR. O’Doherty et al [51] advocate the need for a broader consideration, which does not only rely on governance mechanisms such as informed consent and anonymization, as it tends to focus merely on the individual. We also advocate that further aspects need to be considered when sharing information, such as the provision of information, opportunity to opt out, and a review mechanism. Governance mechanisms that also need to be considered are cyber security technologies (eg, access controls and encryption) to safeguard data, along with fostering greater public involvement, transparency, and democratic discourse about this issue [51]. Information security focusing on consent and anonymization as a legal basis is too narrowly focused; wider societal concerns are not addressed. Hence, they are, as O’Doherty et al [51] state, “insufficient to protect against subversion of health databases for nonsanctioned secondary uses, or to provide guidance for reasonable but controversial secondary uses.” Our results support the finding that if the purposes are of great societal value and not only advertising or marketing, then people find it more acceptable to share their health information. Adding a review process increased the probability of people accepting their health information being shared even further.

Aitken et al [10] investigated public preferences for sharing health information in the context of research. In their study, similar attribute selection was made regarding the identity of the new user, what type of information was shared, the purpose of data sharing, and oversight of the process. In contrast to the study by Aitken et al [10], our study results emphasize the importance of respondents being informed of the new user and further use of health information. The reason that our respondents valued the opportunity to be informed might be due to the scope of our study. Our study includes data sharing between different contexts, whereas the previous study only examined data sharing within the academic research context.

An earlier study indicated that people were moderately happy to share most types of information, with least support for sharing personal information such as marital status, age, and income status [52]. Other studies also showed support for data sharing in medical research, as long as the data are pseudonymized [53,54]. In our study, we asked respondents to assume that all shared health information would be pseudonymized. The reason for sharing became more important when genetic information was shared. If the health information collectors and the new users can ensure that there is a guarantee for people remaining anonymous, and successfully manage to communicate this, it will facilitate data sharing in the future. However, this is not applicable for genetic data, as such data are uniquely identifiable and can generally not be anonymized. However, it might be possible to compensate for this if the reason for using the data can be well communicated.

The results provided a deeper understanding of context-specific factors that people value and provide a robust evidence base, which both confirm and challenge the current policy [55]. We should understand that the willingness to share health information varies depending on contextual properties. In particular, the situation in which information is gathered, who the data is being shared with, for what purpose and whether consent is provided, and the extent to which these preferences change depending on which Northern European country the respondents live in. We hypothesized that respondents would make a different choice depending on the context, and all attributes significantly contributed to the choice. This is in accordance with the theory of contextual integrity [17], which finds that privacy is perceived and expected differently depending on the norms and values surrounding the context. People do not request complete control over information about themselves, or that no information about them should be shared. It is important to note that this is shared appropriately. In Table 6, we can see the different probabilities of respondents accepting to share their data in different scenarios. The reason for sharing plays a major role for respondents, as does the opportunity to opt out or consent. Adding a second governance mechanism would increase the number of people accepting to share their health information.

This is one of the first DCE studies on this topic and is very valuable for ongoing cyber security discussions. However, there might be a hypothetical bias, as in all DCEs. This risk is due to respondents not being bound by their hypothetical choices: they might, in reality, choose something different from what they stated.

This is the first DCE study to compare the preferences of people in Nordic countries for sharing health information. Moderating and mediating factors such as level of education, gender, health status, and e-literacy need further investigation, as they may affect the differences between the countries in preference choices.

In this study, any attribute referring to personal benefits to individuals concerned when health information was shared was excluded. Aitken et al [10] included the attribute *profit-making*, which we considered included in our DCE because of its relative ranking. However, following discussions with the research team and the cognitive interviews, it was excluded because benefit is already incorporated in the nature of some combinations. For example, when a new user is a technological company and the new reason is to develop a new product or service; then, it is understood that the company will benefit financially from the shared health information. Similarly, if health information is shared with health care to evaluate care, it is implicit that both society and individuals benefit. However, whether the actual transfer involves a monetary exchange could still be a relevant attribute in some contexts.

### Conclusions

Taking the public’s acceptance of sharing data into account becomes more important in policy making in the digital world. This study provides insights into the cyber security and privacy research areas on how important specific elements of data sharing are for the public when they consider sharing their data. This is useful for further policy making on the governance of health data in the digital world. At the same time, this provides crucial insights into how to approach people about sharing their data with health care, research projects, national authorities, or different companies. On average, respondents were hesitant to share health information. Respondents’ willingness to share their data was most impacted by giving them information about what would happen with their data and the possibility of opting out. To have a review system in place is important for the respondents. Respondents from the studied countries differed in their preferences for sharing health data. This choice of consent or opt out should be further investigated to meet the challenges of the extensive need to share health data digitally and the heterogeneity in people’s preferences.

## Appendix

Multimedia Appendix 1 The relative importance stratified by the type of health information.

Multimedia Appendix 2 Acceptance uptake (%) when health information is shared from a technological company to a new technological company and the acceptance uptake when health information is shared to develop a new product or service and with no review process.

## Abbreviations

DCE: discrete choice experiment
eHL: eHealth literacy
GDPR: General Data Protection Regulation
