# Supplementary material for: Preferences of the Public for Sharing Health Data: Discrete Choice Experiment
Source: JMIR Med Inform. 2021 Jul 5;9(7):e29614. doi: 10.2196/29614 (PMC8406119; doi:10.2196/29614)

Multimedia Appendix 1.

Relative importance score for respondents' preferences stratified on type of health information. The reason for the new data user was more important for genetics information compared to life-style information.

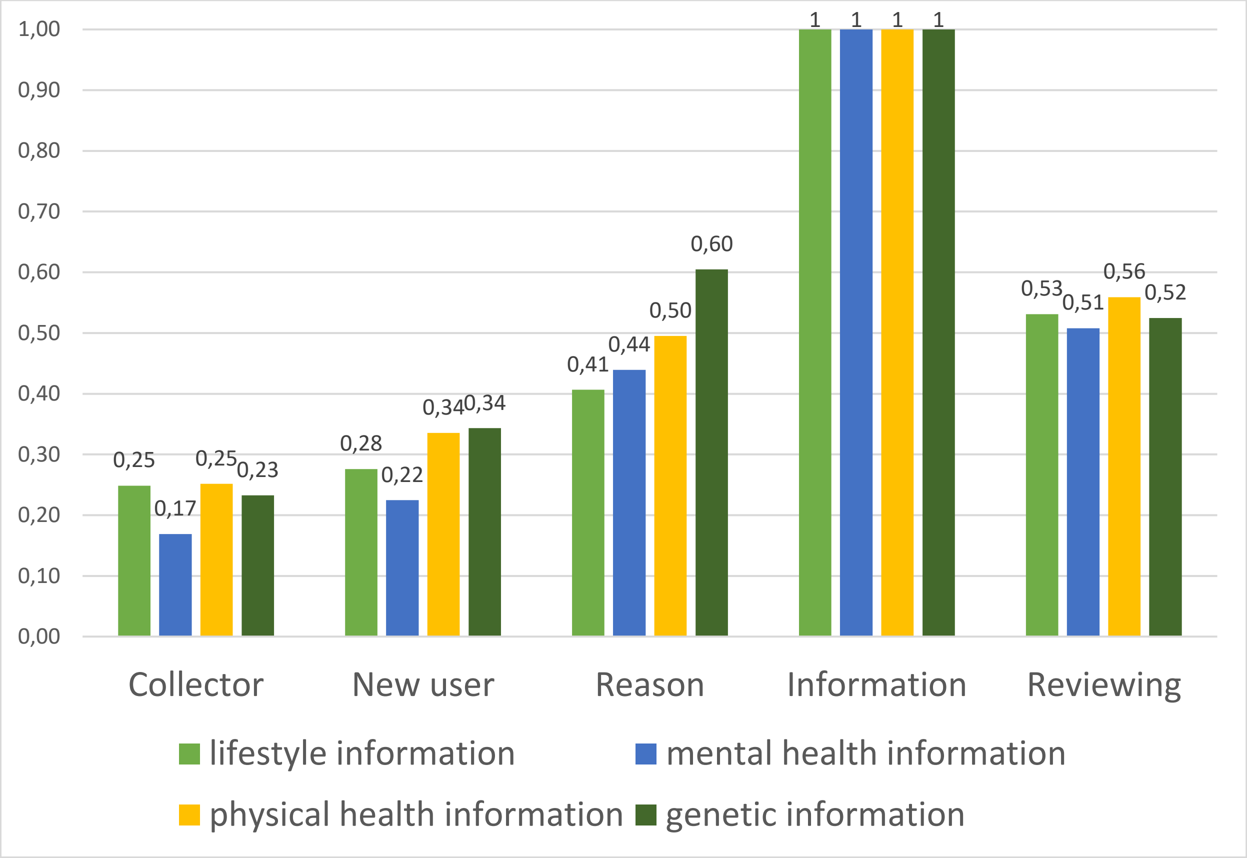

Supplement: Multimedia Appendix 1 [file medinform_v9i7e29614_app1.pdf]
