# Supplementary material for: Preferences of the Public for Sharing Health Data: Discrete Choice Experiment
Source: JMIR Med Inform. 2021 Jul 5;9(7):e29614. doi: 10.2196/29614 (PMC8406119; doi:10.2196/29614)
Supplement: Multimedia Appendix 2 [file medinform_v9i7e29614_app2.pdf]

Multimedia Appendix 2.

The acceptance uptake in percentage when health information is shared from *a technological company* to a *new technological company*.

	**Not informed**, %	**Informed**, %	**Opt out**, %	**Consent**, %
**No review**				
Develop a new product or service	28	43	57	58
Promoting or advertising or marketing	14	31	48	55
Investigate a policy initiative	25	41	56	56
Evaluate the quality	27	44	58	58
**Review of use**				
Develop a new product or service	40	58	71	66
Promoting or advertising or marketing	27	46	60	60
Investigate a policy initiative	38	56	69	68
Evaluate the quality	40	60	71	71
**Review of use and transfer**				
Develop a new product or service	40	58	71	70
Promoting or advertising or marketing	26	46	60	59
Investigate a policy initiative	38	56	69	68
Evaluate the quality	40	60	71	70

The acceptance uptake in percentage when health information is shared to develop *a new product or service* and with *no review process*.

	**Technological company**, %	**Pharmaceutical company**, %	**A research project**, %	**A national authority**, %
**Not informed**				
A technological company	24	31	29	33
A research project	30	37	35	39
Your healthcare provider	35	42	40	44
**Informed**				
A technological company	39	44	43	46
A research project	49	53	55	56
Your healthcare provider	53	57	61	61
**Informed opt-out**				
A technological company	48	52	56	56
A research project	59	63	71	69
Your healthcare provider	63	66	76	74
**Information consent**				
A technological company	46	50	54	54
A research project	57	61	69	68
Your healthcare provider	62	64	75	73
